# Erasmus Syndrome: Diffuse Systemic Sclerosis With Silicosis

**DOI:** 10.7759/cureus.77900

**Published:** 2025-01-24

**Authors:** Rajesh Kumar, Ruthra Kumaran, Shivani Manchem, Chandan Kumar, Manoranjan Sahoo

**Affiliations:** 1 Internal Medicine, All India Institute of Medical Sciences, Deoghar, IND

**Keywords:** coal worker pneumoconiosis, erasmus syndrome, occupational diseases, silica exposure, silicosis, systemic sclerosis

## Abstract

This report describes the case of a 30-year-old man with a history of prolonged occupational exposure to silica and coal dust leading to the development of Erasmus syndrome, a rare condition of silicosis associated with autoimmune disease, i.e., systemic sclerosis, in a short period. The patient was a laborer in the mining and construction industry, and he presented within a decade of occupational exposure with progressive dyspnea, chronic cough, skin thickening, inflammatory polyarthritis, and Raynaud's phenomenon. Clinical, radiological, and serological tests revealed "accelerated silicosis-associated diffuse systemic sclerosis". Treatment involved stoppage of occupational exposure to silica and symptomatic and immunosuppressive therapy.

The case highlights the significance of occupational silica exposure in the development of autoimmune diseases and stresses the need for early intervention, the initiation of protective measures, and the strict monitoring of permissible silica exposure by regulatory authorities.

## Introduction

Erasmus syndrome is a rare occupational disorder associated with prolonged silica exposure leading to the development of silicosis and systemic sclerosis [1,2]. This syndrome is more prevalent in men as they have to work outdoors for their livelihood, especially in workplaces with more exposure to respirable silica and coal dust [3]. While the overall prevalence of Erasmus syndrome is extremely low, it represents an important occupational health hazard because of its propensity to augment the disease progression leading to severe pulmonary involvement, the development of autoimmune diseases like systemic sclerosis [4], increased disability-adjusted life years (DALYs), and even death.

The exact cause of Erasmus syndrome remains unclear, though it is believed to result from the association of silica or its derivatives with cellular and humoral immunity. Exposure to silica dust, hydrocarbons, pesticides, plastics, solvents, and welding fumes has been linked to its pathogenesis [1,4]. The syndrome was first identified in 1957 among gold miners who developed systemic sclerosis after long-term exposure to silica [2,4]. In India, the earliest reported case was documented in 1997 by Khanna et al., emphasizing the relevance of this disorder in occupational settings worldwide [3].

This case highlights the importance of early recognition of Erasmus syndrome and the need for comprehensive occupational safety protocols, including regular screenings, protective measures, and regular surveillance of silica exposure limit. Such proactive interventions are essential to reduce disease progression, improve outcomes, and prevent further occupational menace [1,5].

## Case presentation

A 30-year-old man with no comorbidities presented to our hospital with progressive shortness of breath (change of modified Medical Research Council (mMRC) dyspnea grades 1-4 over five years), chronic dry cough, and skin tightening over the past five years. These symptoms, which developed in less than a decade, were due to exposure to silica and silicates found in coal, in his workplace. He worked in a stone-crushing factory for about four years, in road construction for another three years, and as a shipyard sweeper cleaning coal dust for another four years. There was neither a history of regular use of appropriate protective equipment due to limited availability nor appropriate monitoring measures of respirable silica in his workplace. The patient had a temporary decrease in cough during breaks from work initially, but his symptoms worsened upon returning to work for his livelihood in the same dust-laden environments.

Gradually over the past five years, the patient experienced progressive symmetrically tightening and darkening of the skin of the upper limb spreading proximally from the distal part and face and the development of Raynaud's phenomenon. Additionally, he also had a history of symmetrical inflammatory joint pains in the hand for the last one year. There is also a history of taking six months of empirical anti-tuberculosis therapy from a peripheral health center before his admission to our hospital.

**Figure 1:**
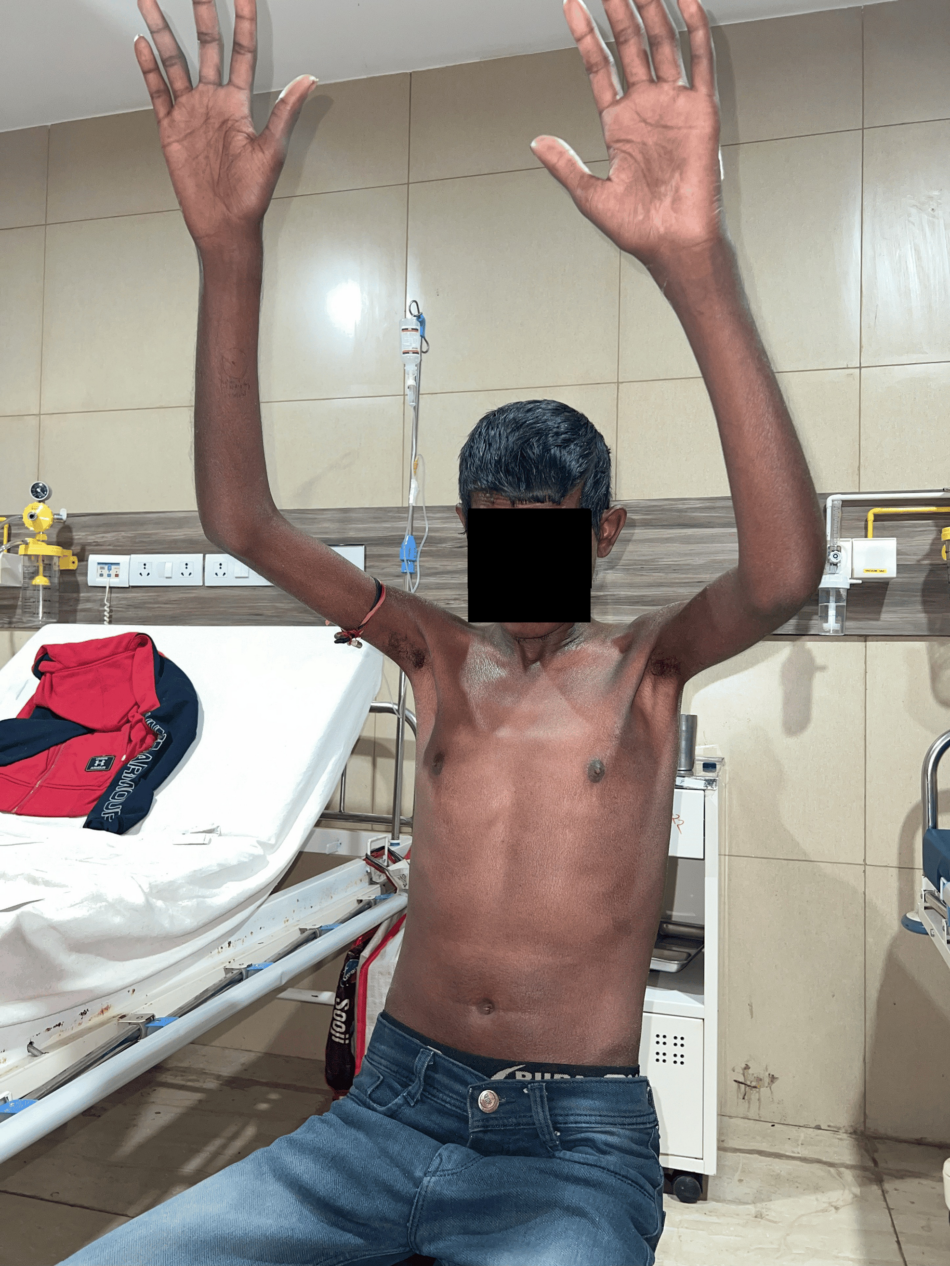
Binding of skin present over the face, proximal and distal extremities, and trunk: mRSS score 25/51 mRSS: modified Rodnan skin score

**Figure 2:**
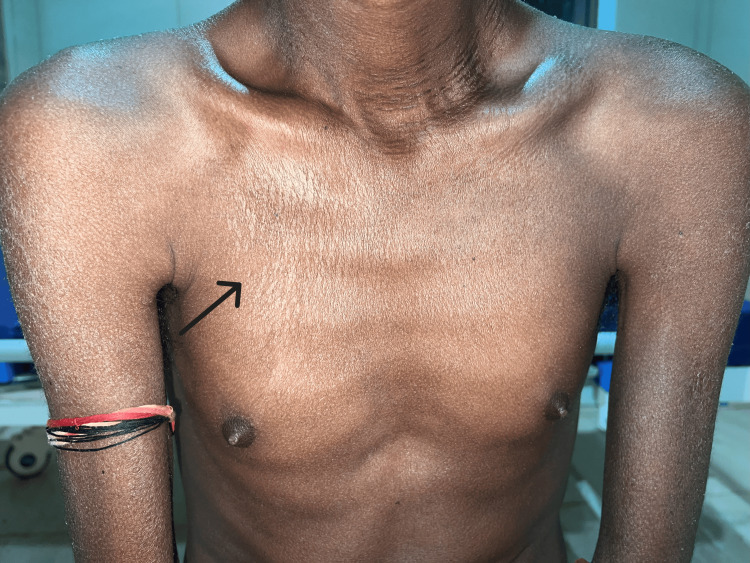
Hyperpigmented and hypopigmented rashes over the chest (salt and pepper pattern)

Upon examination, the skin on the forehead was shiny, exhibiting a noticeable loss of wrinkles and microstomia(Figure 1). Thickened skin was observed on the back and chest, accompanied by hyperpigmented and hypopigmented patches that resembled a "salt and pepper" pattern (Figure 2). Generalized muscular atrophy was noted in both arms and forearms, with fixed flexion deformities present at the elbows and proximal interphalangeal joints. Digital pits were seen on the right and left middle finger. On auscultation, bilateral end-inspiratory crackles were detected in all lung fields with a systolic murmur of tricuspid regurgitation which was audible over the tricuspid area.

On investigations, routine complete blood picture, renal function, liver function test, thyroid profile, and blood glucose level revealed no abnormality. Inflammatory markers such as erythrocyte sedimentation rate (ESR) and C-reactive protein (CRP) were elevated (ESR 50 mm/hr, CRP 30 mg/l). Viral serology was non-reactive. Microbiological sputum analysis (gram stain, AFB (acid-fast bacilli), cartridge-based nucleic acid amplification test (CBNAAT), culture) from bronchial washings ruled out tuberculosis and bacterial and fungal growth. Other tests like rheumatoid factor, serum angiotensin-converting enzymes, and *Aspergillus fumigatus*-specific IgG levels were also within normal range.

Serological testing revealed an antinuclear antibody (ANA) nucleolar pattern (2+ with a 1:80 end titer) and strongly positive anti-Scl-70 antibodies (3+). Nailfold capillaroscopy showed periungual telangiectasia and capillary dropouts. These findings were suggestive of systemic sclerosis.

Pulmonary function testing demonstrated a restrictive pattern (forced expiratory volume in one second (FEV1)/forced vital capacity (FVC) ratio of 95%) and a reduced FVC of 60%. Echocardiography revealed mild pulmonary arterial hypertension and mild tricuspid regurgitation (peak velocity of 3.4 m/s, right ventricular systolic pressure (RVSP) of 42 mm Hg, left ventricular ejection fraction (LVEF) of 55.19%). The diffusing capacity for carbon monoxide (DLCO) was 50% of the predicted value. Lymph node biopsy could not be done as consent was not given by the patient.

**Figure 3:**
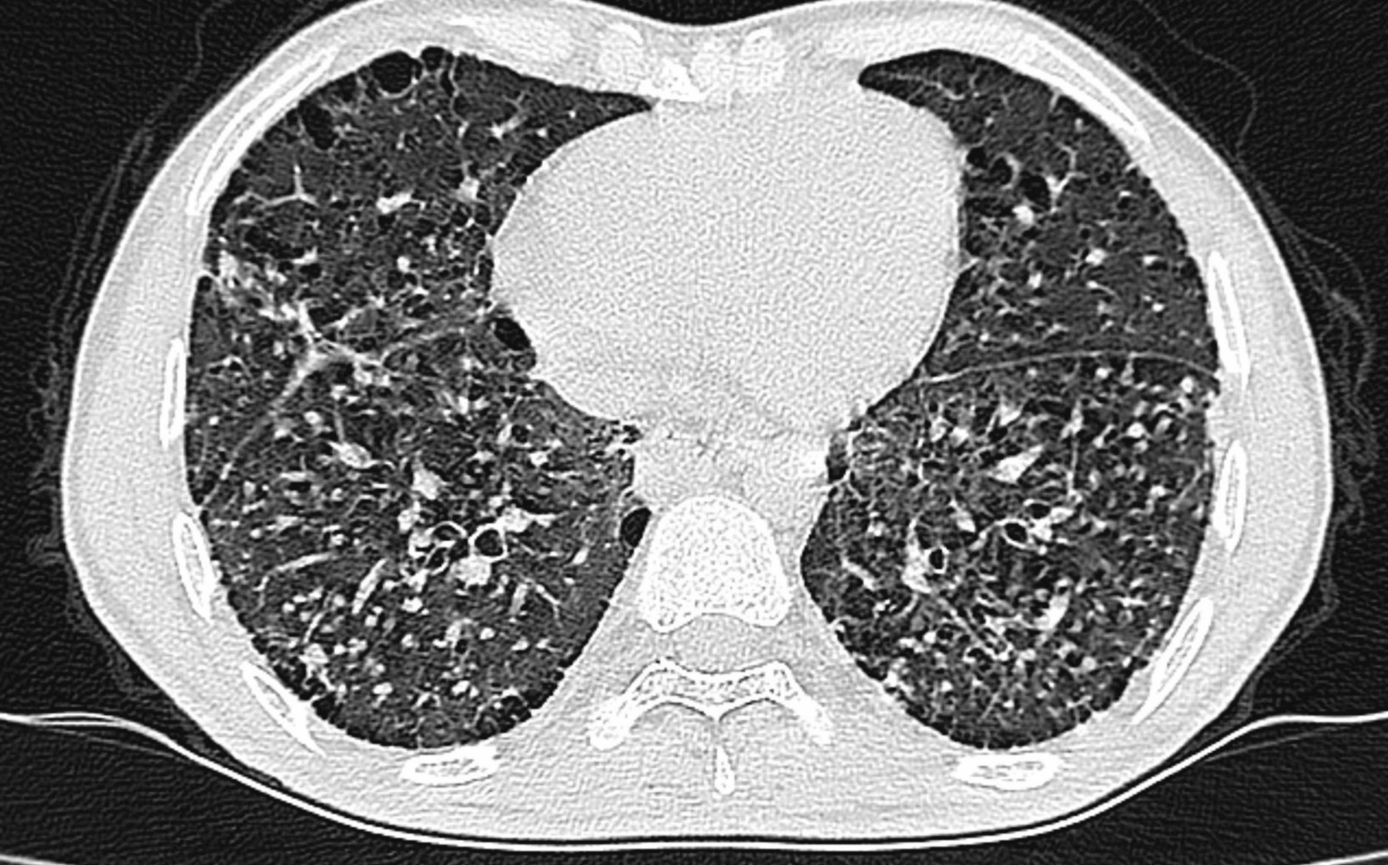
Ground-glass opacification with subpleural sparing and interlobular septal thickening in the lower lobes (NSIP-ILD) NSIP: nonspecific interstitial pneumonia; ILD: interstitial lung disease

**Figure 4:**
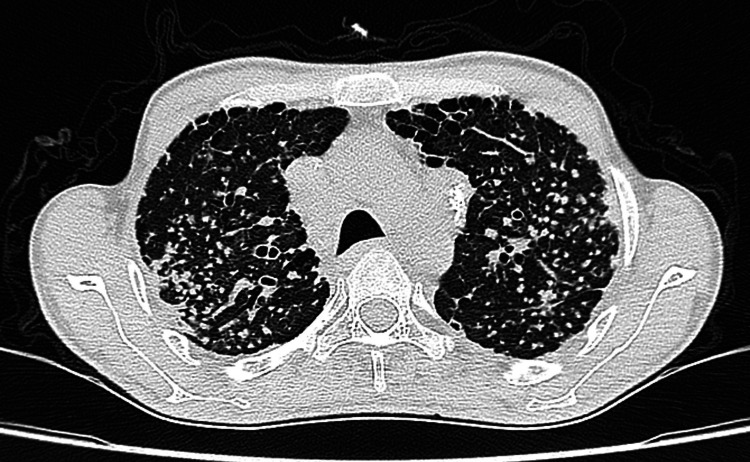
Chest HRCT showing numerous well-defined small nodules noted found in centrilobular distribution, predominately in the upper lobe in both lungs HRCT: high-resolution computed tomography

**Figure 5:**
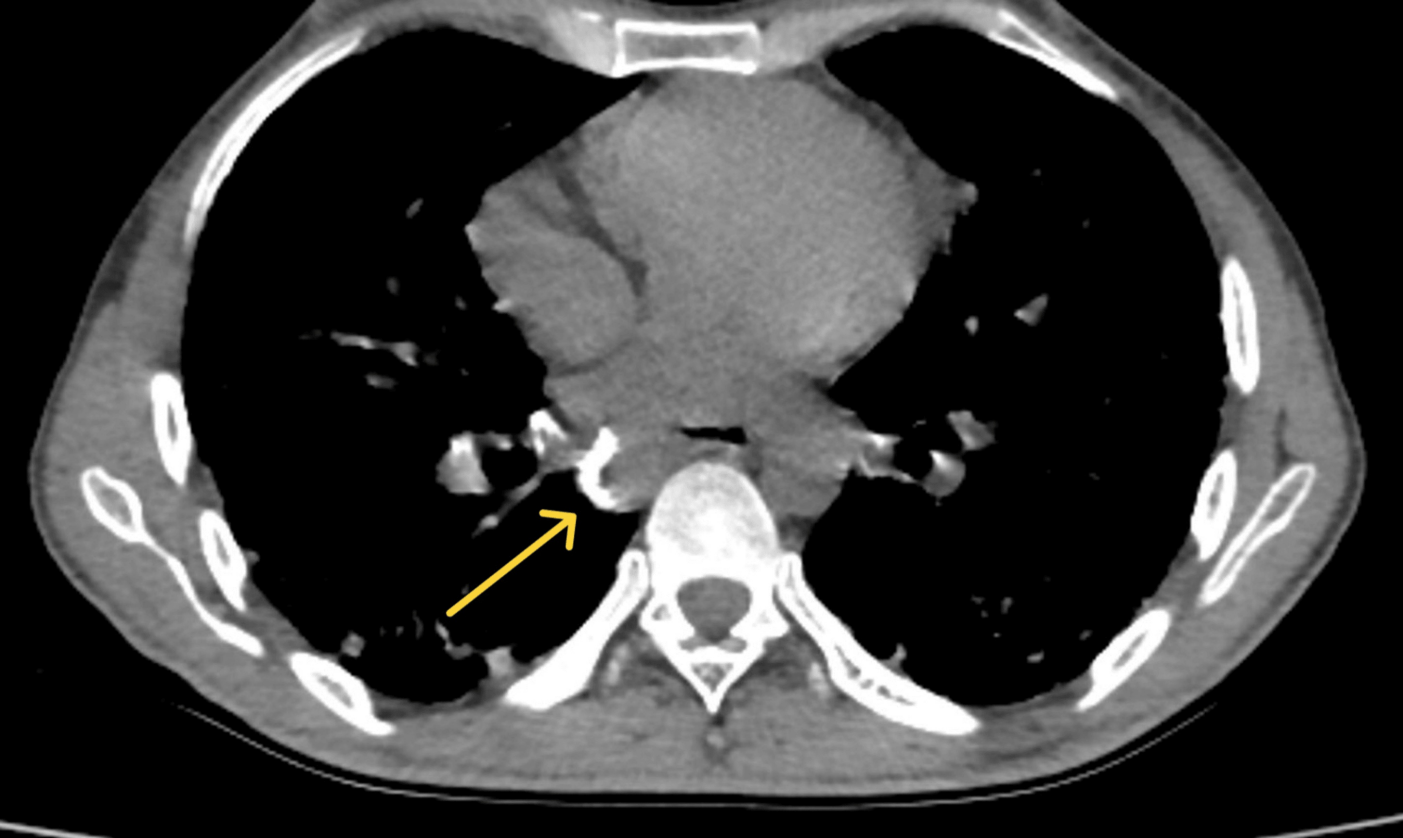
Multiple calcified hilar and mediastinal lymphadenopathy: "eggshell calcification pattern"

Chest high-resolution computed tomography (HRCT) revealed centrilobular nodules (Figure 3), conglomerate opacities (Figure 4)*, *ground-glass opacifications(Figures 3-4) of nonspecific interstitial pneumonitis pattern (cellular nonspecific interstitial pneumonia (NSIP)-interstitial lung disease (ILD)), and peripheral hilar lymph node calcification (Figure 5). These imaging findings were very suggestive of accelerated silicosis with systemic sclerosis-related ILD.

The patient was diagnosed with Erasmus syndrome, a rare case of accelerated silicosis with systemic sclerosis based on the patient's history of occupational exposure to prolonged silica and coal dust, clinical findings (progressive dyspnea and skin thickening, cough, and Raynaud's phenomenon), serological markers (positive anti-Scl-70 antibodies), and imaging studies. In this type of presentation, tuberculosis is an important differential (ruled out here). Other possibilities are sarcoidosis and malignancies. 

Treatment

The patient was advised to avoid silica and coal dust exposure. He was treated with mycophenolate mofetil 2 g/daily (immunosuppressive) and supportive care that included the use of calcium channel blockers for Raynaud's phenomenon (nifedipine), endothelial receptor antagonist (bosentan) for pulmonary hypertension to improve exercise tolerance, and physiotherapy to improve joint mobility. Regular follow-ups were planned to monitor pulmonary function and disease progression.

## Discussion

Erasmus syndrome is a rare occupational rheumatological disorder characterized by the development of systemic sclerosis following exposure to silica, with or without associated silicosis. The exact pathophysiology involves the silica-induced activation of macrophages, which release inflammatory cytokines such as interleukin (IL)-1, IL-2, and tumor necrosis factor (TNF)-alpha [4]. These cytokines stimulate T-helper cells and promote fibroblast activation, resulting in excessive collagen deposition, which is the hallmark of systemic sclerosis [1]. Chronic silica exposure is associated with immune dysregulation, including an increase in soluble IL-2 receptor levels, which further exacerbates the autoimmune response.

This case report highlights the accelerated progression (less than a decade) of diffuse systemic sclerosis in a young man with significant occupational exposure to both silica and coal dust. The patient's clinical presentation, serological findings (positive anti-Scl-70 antibodies), and imaging results (indicative of accelerated silicosis with systemic sclerosis-related NSIP-ILD pattern) were pivotal in confirming the diagnosis. Accelerated silicosis occurs when symptoms start in less than 10 years of exposure along with typical chest HRCT findings (acute silicosis occurs in weeks to five years of exposure; chronic silicosis occurs in 10-30 years of exposure). Chest HRCT findings in acute silicosis have an alveolar filling, whereas, in chronic silicosis, progressive massive fibrosis is found.

Although idiopathic systemic sclerosis and silica-associated systemic sclerosis share similar clinical features, the latter is more prevalent in men due to occupational exposure and tends to have severe pulmonary manifestations. A meta-analysis conducted between 2012 and 2022 revealed that Erasmus syndrome most commonly affects men, with an average onset age of 51.7 years, following chronic silica exposure ranging from six to 47 years [5]. In contrast, our patient developed systemic sclerosis at a younger age, beginning occupational exposure at 15 years and presenting with symptoms by 25 years [6]. The rapid progression of the disease in this case is likely due to the combined effects of silica and coal dust exposure [5]. Rubio-Rivas et al. also emphasized the higher prevalence of severe pulmonary involvement in silica-associated systemic sclerosis compared to idiopathic forms [1]. Similarly, Jain et al. documented a case that focuses on the importance of occupational history in diagnosing systemic sclerosis, highlighting the occupational links that often underlie disease progression [6]. Current guidelines for managing systemic sclerosis, including those associated with occupational exposure, emphasize a multidisciplinary approach [7]. This includes symptomatic management, immunosuppressive therapy, and preventive measures such as occupational safety protocols. Early intervention is crucial to mitigate complications, particularly pulmonary hypertension and ILD, which are major contributors to morbidity and mortality.

This case underscores the need for stringent occupational health regulations to minimize exposure to silica and coal dust as well as the importance of considering occupational history in autoimmune diseases.

## Conclusions

Occupational exposure to silica and coal dust is a significant risk factor for the development of systemic sclerosis, known as Erasmus syndrome, which can progress rapidly. Early recognition of systemic sclerosis in patients with occupational exposure can prevent severe complications through timely intervention and cessation of exposure. Detailed occupational history is critical in the differential diagnosis of systemic sclerosis, especially in young men presenting with pulmonary and skin manifestations. Silica-associated systemic sclerosis often exhibits more severe pulmonary involvement compared to idiopathic forms, necessitating targeted diagnostic and therapeutic strategies. Protective measures, including the use of personal protective equipment and routine health screenings in high-risk occupations, are essential to prevent silica-associated autoimmune disorders.

## References

[1] Rubio-Rivas M, Moreno R, Corbella X (2017). Occupational and environmental scleroderma. Systematic review and meta-analysis. Clin Rheumatol.

[2] Erasmus LD (1957). Scleroderma in goldminers on the Witwatersrand with particular reference to pulmonary manifestations. S Afr J Lab Clin Med.

[3] Khanna N, D'Souza P, Sud A, Pandhi RK (1997). Systemic sclerosis in a stone cutter. Indian J Dermatol Venereol Leprol.

[4] Stansbury RC, Petsonk EL, Parker JE (2015). Coal workers' lung diseases and silicosis. Fishman's Pulmonary Diseases and Disorders.

[5] Lomanta JM, Atienza MA, Gonzales JR, Amante EJ, Urquiza SC, Lucero-Orillaza H, Santiaguel JM (2022). Erasmus syndrome: a case report and literature review. Am J Case Rep.

[6] Jain S, Joshi V, Rathore YS, Khippal N (2017). Erasmus syndrome: silicosis and systemic sclerosis. Indian J Occup Environ Med.

[7] Freire M, Alonso M, Rivera A (2015). Clinical peculiarities of patients with scleroderma exposed to silica: a systematic review of the literature. Semin Arthritis Rheum.

